# Whole-cell response to nitrogen deprivation in the diatom *Phaeodactylum tricornutum*

**DOI:** 10.1093/jxb/erv340

**Published:** 2015-07-10

**Authors:** Leila Alipanah, Jens Rohloff, Per Winge, Atle M. Bones, Tore Brembu

**Affiliations:** Department of Biology, Norwegian University of Science and Technology, N-7491 Trondheim, Norway

**Keywords:** Carbon metabolism, diatom, metabolomics, nitrogen deprivation, *Phaeodactylum tricornutum*, transcriptome, triacylglycerol.

## Abstract

Algal growth is strongly affected by nitrogen (N) availability. Diatoms, an ecologically important group of unicellular algae, have evolved several acclimation mechanisms to cope with N deprivation. In this study, we integrated physiological data with transcriptional and metabolite data to reveal molecular and metabolic modifications in N-deprived conditions in the marine diatom *Phaeodactylum tricornutum*. Physiological and metabolite measurements indicated that the photosynthetic capacity and chlorophyll content of the cells decreased, while neutral lipids increased in N-deprived cultures. Global gene expression analysis showed that *P. tricornutum* responded to N deprivation through an increase in N transport, assimilation, and utilization of organic N resources. Following N deprivation, reduced biosynthesis and increased recycling of N compounds like amino acids, proteins, and nucleic acids was observed at the transcript level. The majority of the genes associated with photosynthesis and chlorophyll biosynthesis were also repressed. Carbon metabolism was restructured through downregulation of the Calvin cycle and chrysolaminarin biosynthesis, and co-ordinated upregulation of glycolysis, the tricarboxylic acid cycle, and pyruvate metabolism, leading to funnelling of carbon sources to lipid metabolism. Finally, reallocation of membrane lipids and induction of *de novo* triacylglycerol biosynthesis directed cells to accumulation of neutral lipids.

## Highlight

An integrated transcriptomics and metabolomics analysis of the nitrogen-deprivation response in the diatom *Phaeodactylum tricornutum* uncovered remobilization of internal nitrogen-containing resources, and remodelling of carbon and lipid structures.

## Introduction

Phytoplankton blooms vary temporally and spatially in accordance with nutrient availability (Brandes *et al.*, 2007; Falkowski and Raven, 2007). Under upwelling conditions, high levels of available nitrate and iron lead to an increase in phytoplankton biomass, which is generally dominated by diatoms (Kudela and Dugdale, 2000; Capone and Hutchins, 2013). Inorganic nitrogen (N) in the form of ammonia or nitrate is utilized by several phytoplankton (Dham *et al.*, 2005); some phytoplankton are also able to use organic forms of nitrogen such as amino acids, nucleic acids, and urea (Baker *et al.*, 2009; Solomon *et al.*, 2010).

Diatoms are a group of unicellular heterokont microalgae believed to include some 200 000 species (Armbrust, 2009). It is estimated that marine diatoms are responsible for about 32% of global phytoplankton primary production (Uitz *et al.*, 2010). Unlike plants and green algae, diatoms and other heterokonts originate from a serial secondary endosymbiosis event, in which a green alga and subsequently a red alga were engulfed by a heterotrophic eukaryote (Moustafa *et al.*, 2009; Bowler *et al.*, 2010). In addition, a large number of horizontal gene transfer events have further increased the gene repertoire. As a result, diatom genomes contain unique combinations of nutrient assimilation and metabolic pathways that have contributed to their ecological success in the ocean (Prihoda *et al.*, 2012).

The whole-genome sequences of the centric diatom *Thalassiosira pseudonana* (Armbrust *et al.*, 2004) and the pennate diatom *Phaeodactylum tricornutum* (Bowler *et al.*, 2008) have provided valuable information on the regulatory and metabolic inventory of these diatoms. The genomes of *T. pseudonana* and *P. tricornutum* contain several transporter proteins for uptake of inorganic and organic nitrogen (Rees and Syrett, 1979; Armbrust *et al.*, 2004; Allen, 2005; Hildebrand, 2005; Solomon *et al.*, 2010). Nitrate entering the cell is first reduced to nitrite and ammonium (Allen *et al.*, 2005; Bowler *et al.*, 2010). Ammonium is then assimilated by glutamate synthase/glutamine synthetase to amino acids (Zehr and Falkowski, 1988; Takabayashi *et al.*, 2005). Diatoms possess plastidial glutamine synthetase (GSII) and glutamate synthase (Fd-GOGAT) as well as mitochondrial NAD(P)H-GOGAT and GSIII (Bowler *et al.*, 2010; Allen et al., 2011). Mitochondrial GSIII may catalyse the assimilation of glutamine from ammonium derived from cytosolic catabolic reactions, e.g. deamination and hydrolysis of organic N (Hockin *et al.*, 2012; Kissen *et al.*, 2010; Parker and Armbrust, 2005).

N and carbon metabolism are closely connected to each other. N assimilation and amino acid biosynthesis require reducing equivalents from photosynthesis and carbon skeletons from the tricarboxylic acid (TCA) cycle (Hockin *et al.*, 2012). Moreover, in the photosynthetic apparatus, assimilated N is used for example in ribulose-1,5-bisphosphate carboxylase (Rubisco) and the light-harvesting complex (LHC) (Orellana and Perry, 1995; Foyer *et al.*, 2003; Nunes-Nesi *et al.*, 2010). In response to N deprivation, diatoms reprogram several metabolic pathways. The impact of N deprivation on pigments, photosynthesis, carbon fixation, and N assimilation has been studied in diatoms (Syrett *et al.*, 1986; Kolber *et al.*, 1988; Geider *et al.*, 1993; Granum *et al.*, 2009; Bender *et al.*, 2014). Diatoms store carbon in the form of 1,3-β-d-glucan (chrysolaminarin) or lipids (Kroth *et al.*, 2008). Under optimal conditions, chrysolaminarin is the major sink of carbon storage in the vacuoles (Granum and Myklestad, 2002). Under several stress conditions, in particular N starvation, diatoms change their carbon storage patterns in favour of neutral lipid accumulation (Eizadora *et al.*, 2009; Norici *et al.*, 2011; Sharma *et al.*, 2012; Valenzuela *et al.*, 2012). Neutral lipids produced from microalgae have been proposed as a sustainable substitute biofuel for fossil fuels (Wijffels and Barbosa, 2010). Other N-containing compounds, such as proteins and nucleic acids, are also affected by a decrease in cellular N content (Olson *et al.*, 1986; Mock and Kroon, 2002; Bertozzini *et al.*, 2013; Mus *et al.*, 2013).

To understand how the oleaginous marine diatom *P. tricornutum* responds to N deprivation, cells were grown in f/2 medium and in N-free medium, and samplings were conducted at 48 and 72h after N deprivation. We combined transcriptional and metabolite analyses to monitor the effect of N deprivation at different molecular levels in order to get a better insight into the acclimation strategies employed by *P. tricornutum* under N deprivation. These data were further complemented by physiological data such as measurements of cell growth, neutral lipids, and other cell chemistry measurements. We use this data to predict metabolic changes in N-deprived cells leading to remodelling of lipid metabolism and triacylglycerol (TAG) accumulation.

## Materials and methods

### Growth conditions and treatments

Axenic cultures of *P. tricornutum* clone Pt1 8.6 (CCMP632) were grown in f/2 medium and kept in exponential growth at 15 °C under continuous white fluorescent light (60 µmol photons m^–2^ s^–1^) for 3 weeks. Bacterial contamination was checked regularly by inoculation in peptone-enriched f/2 medium (Andersen *et al.*, 1997). Growth medium (f/2) was made from 0.2 µm-filtered seawater, autoclaved, and enriched with macro- and micronutrients (Guillard, 1975). Three or four replicates of the start culture (6–7ml) were transferred to 220ml of medium supplemented with complete f/2 nutrients (replete) or f/2 without added nitrate (deprived). The nitrate concentration in the seawater used for the experiments was measured to 10 μM, which is 1.1% of the f/2 nitrate concentration. Cells were incubated in batch cultures with a starting cell density of 5×10^4^ ml^–1^ in sterile culture flasks with a 75cm^2^ growth area. Cell counting and maximum quantum yield of photosystem II (PSII) (F_v_/F_m_) was measured daily using a Bürker–Türk counting chamber and AquaPen-C AP-C 100 (Photon Systems Instruments), respectively. For the other experiments, samples were harvested 48 and 72h after the beginning of the treatment. Samples for RNA and metabolite analysis were stored at –80 °C, while samples for nutrient and pigment analysis were stored at –23 °C until analysis.

### Nutrient analysis

Triplicate cultures for particulate N, carbon, and phosphorus analysis were collected on pre-combusted GF/F filters (particulate C and N analysis) or 0.2 µm GF/F filters (particulate phosphorus), and the flowthrough was used for detection of medium phosphate and nitrate concentration. Triplicate samples for particulate N and carbon analysis along with blank filters were treated with HCl vapour (37%), packed in tin capsules, dried for 2 days at 60 °C, and analysed by an ECS 4010 element analyser (Costech Instruments). All these processes were performed according to Chauton *et al.* (2013). Inorganic nutrients were measured in the filtrate. $NO_{3}^{–}$ + $NO_{2}^{–}$ and $PO_{4}^{3–}$ were analysed in parallel according to I.O. Analytical cartridge Part A002603 and A002604, respectively, as described by Hansen and Koroleff (1999). Particulate phosphate was first oxidized to $PO_{4}^{3–}\;$ according to Norwegian standard NS4725, and then analysed as inorganic $PO_{4}^{3–}$ .

### Pigment analysis

Pigment analysis (fg per cell) was performed based on the protocol by Rodríguez *et al.* (2006). Briefly, 60ml (N replete) or 100ml (N deprived) of cultures was collected on GF/F filters. The cells were extracted with 6ml of 100% ethanol, and extracts were filtered through Millipore 0.45 μm filters. A volume of 73 µl of the final extracts was mixed with 23 µl of water and injected into a Hewlett-Packard HPLC 1100 Series system. Pigments were separated on a Waters Symmetry C_8_ column using the high-performance liquid chromatography (HPLC) method of Zapata *et al.* (2000). Chlorophyll *a* and fucoxanthin were detected by absorbance at 440nm and identified by a diode array detector (λ=350–370nm, 1.2nm spectral resolution). Standard curves were made by isolating pigments separated by HPLC, verifying their identity and quantifying on a spectrophotometer, and running a dilution series on the HPLC instrument. The specific extinction coefficients (α: 1g^–1^ cm^–1^) provided by Egeland *et al.* (2011) were used for pigment quantification.

### Neutral lipid measurement

A volume of 1ml of culture was stained with 1 µl of 0.1 µg ml^–1^ of BODIPY 505/515 (4,4-difluoro-1,3,5,7-tetramethyl-4-bora-3a-4a-diaza-s-indancene; Life Technologies) dissolved in 2% (w/v) dimethyl sulfoxide and shaken carefully by hand (Govender *et al.*, 2012). After 5min, 30 µl of culture was transferred to a microscope slide, and a coverslip was placed on top of the culture and sealed using dental wax. At least 20 cells from two replicates were analysed for BODIPY 505/515 fluorescence on a Leica TCS SP5 confocal laser scanning microscope using a ×63 water objective. *Z*-Sectional images were made using argon laser excitation at 488nm (17% of maximal intensity), and emission was detected with a spectral detector set from 495 to 550nm. Non-confocal bright-field images were made simultaneously. A *z*-stack consisting of 10 scans was made for each cell, encompassing the complete fluorescent part of the cell. The length of the *z*-stack varied between 4.00 and 5.78 μm; consequently, the *z*-slice step size varied between 0.44 and 0.64 μm. Laser power, PMT gain, and offset were kept constant for all scans. Image stacks containing the fluorescence channel were imported into ImageJ (Abràmoff *et al.*, 2004). To determine the total fluorescence detected in the *z*-stack, a region was drawn around each cell to be measured, and three regions next to the selected cell that had no fluorescence were used for background subtraction. The corrected total cell fluorescence for each cell was calculated using the following formula (Gavet and Pines, 2010; Potapova *et al.*, 2011):

$$\begin{array}{l} \text{Whole-cell signal corrected}=\text{whole-cell signal}\\ \;-\;\left(\begin{array}{l} \text{area of selected cell}\times \text{mean }\\ \text{fluorescence of background} \end{array}\right) \end{array}$$

Background fluorescence, as measured from five *z*-sectional images of unstained cells, was negligible (<1% of stained N-replete cells). A total of 20–30 cells were analysed for each treatment.

### Harvesting and extraction for metabolite profiling

Depending on cell density, 60–100ml of culture was collected on 0.65 µm Durapore membrane filters, washed off the filter using 1ml of f/2 medium (N-deprived cells were washed with f/2 without nitrate supplement), and centrifuged at 13 000rpm for 1min at 4 °C. Care was taken to minimize the harvesting time, which was less than 3min. The supernatant was removed and pellets were flash frozen in liquid N_2_ and stored at–80 °C. Metabolites were extracted by adding 1ml of a pre-cooled water:methanol:chloroform (1:2.5:1) mixture containing ribitol as an internal standard (100 µg ml^–1^). Samples were treated for 60min at 60 °C in an ultrasonic bath, centrifuged for 10min at 13 000rpm, and 600 µl aliquots of supernatant were transferred to 2ml Eppendorf tubes.

### Sample derivatization and gas chromatography/mass spectrometry (GC-MS) analysis

Samples were dried in a Savant^TM^ SpeedVac plus SC210A (Thermo Scientific) overnight and stored at –80 °C before derivatization. Dried samples were redissolved in 80 µl of methoxyamine hydrochloride in pyridine (20mg ml^–1^), derivatized for 90min at 30 °C, further treated with 80 µl of *N*-methyl-*N*-(trimethylsilyl)trifluoroacetamide for 30min at 37 °C, and finally transferred to 1.5ml autosampler vials with glass inserts prior to GC-MS. Separations were performed on an Agilent 6890/5975 GC-MS (Agilent Technologies) equipped with a HP-5MS capillary column (30 m×0.25mm internal diameter, film thickness 0.25 µm) (Agilent Technologies). Sample volumes of 3 µl were injected with a split ratio of 15:1. Injection and interface temperature were set to 230 and 250 °C, respectively. The GC temperature program was held isothermically at 70 °C for 5min, ramped from 70 to 310 °C at 5 °C min^–1^, and finally held at 310 °C for 7min (run time: 60min). The MS source was adjusted to 230 °C and a mass range of *m*/*z* 70–600 was recorded (EI mode).

### Metabolite data analysis

Chromatogram visualization and peak identification was carried out using Agilent ChemStation software (Agilent Technologies), AMDIS software (version 2.71; National Institute of Standards and Technology), and OpenChrom Community Edition Synge (version 0.6.0) (Peter Wenig; http://www.openchrom.net). NIST05 spectral library (National Institute of Standards and Technology) in combination with a metabolite target library (Hummel *et al.*, 2010) were used for tentative compound identification. GC-MS data integration, normalization (total signal), and alignment were carried out using the MetAlign software (PRI-Rikilt). Based on distinct quantifier ions, detected metabolites were quantified using the internal standard ribitol (normalized response) and finally expressed in ng per 10^6^ cells. Statistical analysis was carried out using one-way analysis of variance across all time points and N conditions.

A total of 119 metabolites and metabolite tags were detected, 110 of which were tentatively identified based on the MS library. Based on the extraction properties of the solvent mixture, a broad range of metabolites were simultaneously extracted (Nappo *et al.*, 2009), including lipophilic alkanes, fatty acids, and glycerides, and polar compounds such as amino acids, organic acids, sugars, and polyols. A total of 94 metabolites are presented in Supplementary Table S1 (available at *JXB* online), showing the ratio of compound levels in N-deprived cultures to replete conditions after 48 and 72h.

### RNA isolation

Depending on cell density, 60–100ml of cultures was collected on 0.65 µm Durapore membrane filters, washed off the filter using 1ml of f/2 medium (N-deprived cells were washed with f/2 without nitrate supplement), and centrifuged at 13 000rpm for 1min at 4 °C. The supernatant was removed and pellets were flash frozen in liquid N_2_ and stored at –80 °C. Frozen samples were homogenized using a TissueLyser system (Qiagen) for 2×2min at 25 Hz. The samples were placed in a pre-cooled (–80 °C) adapter set for the first shaking step. Before the second shaking step, the samples were transferred to a room temperate adapter set, and 0.5ml of lysis buffer (Spectrum^TM^ Plant Total RNA kit; Sigma-Aldrich) was added to each tube. Total RNA was isolated with a Spectrum^TM^ Plant Total RNA kit (Sigma-Aldrich). To eliminate genomic DNA, an on-column digestion was performed using an RNAase-free DNase I set (Qiagen). Total RNA was quantified using a NanoDrop ND-1000 Spectrophotometer (NanoDrop Technologies). The RNA quality was verified using formaldehyde gel electrophoresis. In addition, RNA integrity was checked on a 2100 Bioanalyzer (Agilent). All samples had RNA integrity numbers above 7.

### cDNA microarray experiments

Total RNA (200ng) was reverse transcribed, amplified, and labelled according to a Low Input Quick Amp Labeling Kit, One-Color (Agilent Technologies). A total of 1650ng of cRNA from each sample was fragmented and hybridized with a Gene Expression Hybridization Kit (Agilent Technologies) on 4×44K *P. tricornutum* whole-genome 60-mer oligonucleotide microarrays (Agilent Technologies) in an Agilent G2545A Hybridization Rotary Oven at 10rpm, 65 °C for 17.5h. Slides were washed with washing buffer 1 and 2 using a Gene Expression Wash Buffer Kit (Agilent Technologies) and directly scanned using a laser scanner (G2505 B; Agilent Technologies) based on the ‘dynamic range expander’ option in the scanner software. Images were processed by Agilent Feature Extraction software version 9.5.

### Statistical analysis

The Limma package (version 3.20.1) (Smyth, 2005) and R version 3.0.3 were used for statistical analysis and identification of significant differentially expressed genes. Single-colour feature expression files from the Agilent microarray scans were imported, and spots identified as feature outliers were excluded from the analysis. Weak or undetected spots were given reduced weight. The data were normalized using the quantile method, and no background subtraction was performed. A design matrix was created and pair-wise comparisons between the samples, DN48 (nitrogen-deprived 48h) and R48 (replete 48h) and DN72 (nitrogen-deprived 72h) and R72 (replete 72h) were performed. The method of Benjamini and Hochberg (1995) was used to estimate the false discovery rate. Genes with an adjusted *P* value of <0.05 were regarded as significantly differentially expressed and were included in the analysis if all oligonucleotides for each gene had a mean adjusted *P* value of <0.05. The study is MIAME compliant. Raw data has been deposited in GEO (accession no. GSE58946).

The Gene Ontology (GO) dataset for biological process was downloaded from the *P. tricornutum* database at Joint Genome Institute (http://genome.jgi-psf.org/Phatr2/Phatr2.home.html). GO terms assigned to significantly regulated genes at each time point were listed separately for up- and downregulated genes. Metabolic pathways were analysed using the DiatomCyc database (Fabris *et al.*, 2012).

### cDNA synthesis and quantitative real-time PCR

cDNA synthesis was performed using 1 µg of total RNA with a QuantiTect Reverse Transcription Kit (Qiagen) following the manufacturer’s instructions. cDNA samples were diluted five times in ddH_2_O before use for quantitative real-time PCR (qRT-PCR) analysis.

Three biological replicates from all treatments were used to perform qRT-PCR on a LightCycler 480 using a LightCycler 480 SYBR Green I Master kit (Roche Applied Science), with a program comprising pre-incubation for 5min at 95 °C, followed by 50 cycles of amplification consisting of 10 s at 95 °C, 10 s at 55 °C, and 10 s at 72 °C. Primer sequences used in the qRT-PCR experiment are given in Table 1. The microarray dataset was screened for genes that were non-responsive to N deprivation at both time points. Based on this screen, Exportin1 (Phatr2_24186) and Aureochrome1 (Phatr2_8113) were selected as reference genes for the qRT-PCR analysis. PCR efficiencies and *C*_t_ values were calculated by linear regression using the LinRegPCR software (Ramakers *et al.*, 2003; Ruijter *et al.*, 2009), and the mean PCR efficiency was calculated for each primer pair. PCR efficiencies and *C*_t_ values were used in the REST 2009 software (Pfaffl *et al.*, 2002) to calculate the statistical significance of difference in expression levels in various treatments. The target genes were normalized to the reference genes in the REST 2009 software.

**Table 1.** Genes analysed by real-time qPCR and their respective primers

Phatr2 ID	Accession	Description	Orientation	Sequence (5′→3′)	Amplicon size (bp)
54101	XP_002177983	Nitrate transporter	Forward	GGAATACTTGCTGTTCCTATGC	58
			Reverse	AGGAGACTCTAGGCTTCGATCT	
34373	XP_002178768	Molybdopterin biosynthesis-like protein CNX5	Forward	ATGCTCAAGGACCGATGCAAAC	130
			Reverse	CAGCTTGGTTCTTACGTCAACA	
17344	XP_002176623	Adenine/guanine permease	Forward	AACTTTACCAGCGATCTTTCGG	87
			Reverse	CTAGAAGAGTACCGCTTGTATC	
18049	XP_002177871	LHC protein LHCF1	Forward	GCAACAACTACCTCGACTTTGG	82
			Reverse	TCCCTGGTTGAGTTCGATAGCA	
49339	XP_002183906	Pyruvate carboxylase PYC2	Forward	GTGGAACTCGTTTCTATCCAAG	116
			Reverse	CGAATCTCCTAACAAGTTCTGG	
24186	XP_002185483	Exportin 1-like protein XPO1	Forward	TCTATTGTTTGGGCGATGAAGC	89
			Reverse	CTTACCGACATTAACCAGCAGT	
8113	XP_002183783	Aureochrome AUREO1a	Forward	GGCTTTCTCAACTTGACGGGAT	116
			Reverse	TTCAATGGCCTTACGGATACGC	

## Results

### Effect of deprived levels of N on physiological responses in *P. tricornutum*

**Fig. 1.**
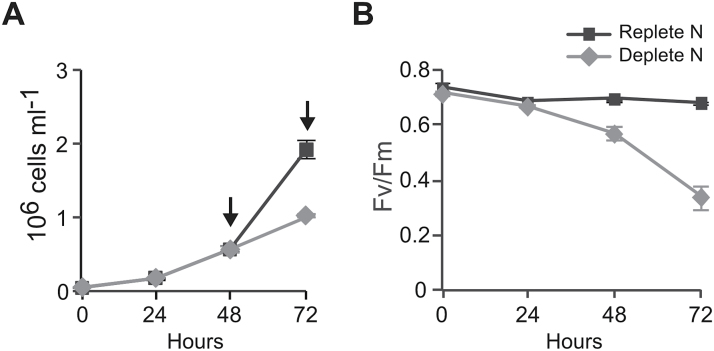
Physiological responses of *P. tricornutum* to nitrate deprivation. Growth curves (A) and changes in maximum quantum yield (F_v_/F_m_) (B) of *P. tricornutum* in N-replete (f/2 medium) and N-deprived (f/2 medium minus nitrate) cultures. Arrows indicate sampling time points. Values are means±standard deviation of four biological replicates.

*P. tricornutum* cell growth was monitored daily in both cultures. All N-replete cultures remained in the exponential phase throughout the time course of the experiment. During the experiment period, cell density increased from 5×10^4^ to 1.92×10^6^ cells ml^–1^ in replete cultures (Fig. 1A). N-free cultures showed similar cell growth compared with N-replete cultures until 48h, but significantly lower growth at 72h, with a cell density of 1.02×10^6^ cells ml^–1^. In order to compare physiological and transcriptional responses before and after the N deprivation started to affect cell growth, the time points of 48 and 72h were chosen for further physiological and molecular experiments.

Nutrient assays of dissolved inorganic nitrate and phosphate demonstrated that none of the replete cultures encountered any deprivation in dissolved inorganic nitrate or phosphate during the whole experiment (Table 2). An increase in the C:N ratio was observed at both time points in N-deprived cells. In N-deprived cells, C:N deviated from the Redfield ratio (Redfield, 1934). The N:P ratio in replete cells was well below the Redfield ratio, but the cultures were still in exponential phase. Reduction of N:P in deprived cells coincided with nitrate loss in the medium.

**Table 2.** Changes in chemical composition, medium nutrient concentration, and pigment concentration of nitrogen-replete (N+) and nitrogen-deprived (N–) cultures 48 and 72h after N deprivation (n=4)

	**N+ 48 h**	**N– 48 h**	**N+ 72 h**	**N– 72 h**
**Cellular nutrient content**				
µg C: µg N	6.03±0.32	9.46±0.73	5.44±0.19	14.65±0.77
µg N: µg P	4.24±0.26	2.37±0.31	4.89±0.18	1.67±0.1
**Medium concentration**				
µg PO_4_^3-^ /l	612,77±2,39	618±2,24	316,99±26,73	576,72±31,38
µg (NO_3_^-^+NO_2_^-^) /l	8797,38±49,23	3,98±1,07	7550,87±108,79	3,8±0,7
**Pigment concentration**				
fg Chl *a* cell^-1^	271.4±25.89	153.2±5.96	271.6±26.29	76.9±9.42
fg fucoxanthin cell^-1^	100.7±3.21	56.8±2.25	96.2±7.73	32.4±3.98

**Fig. 2.**
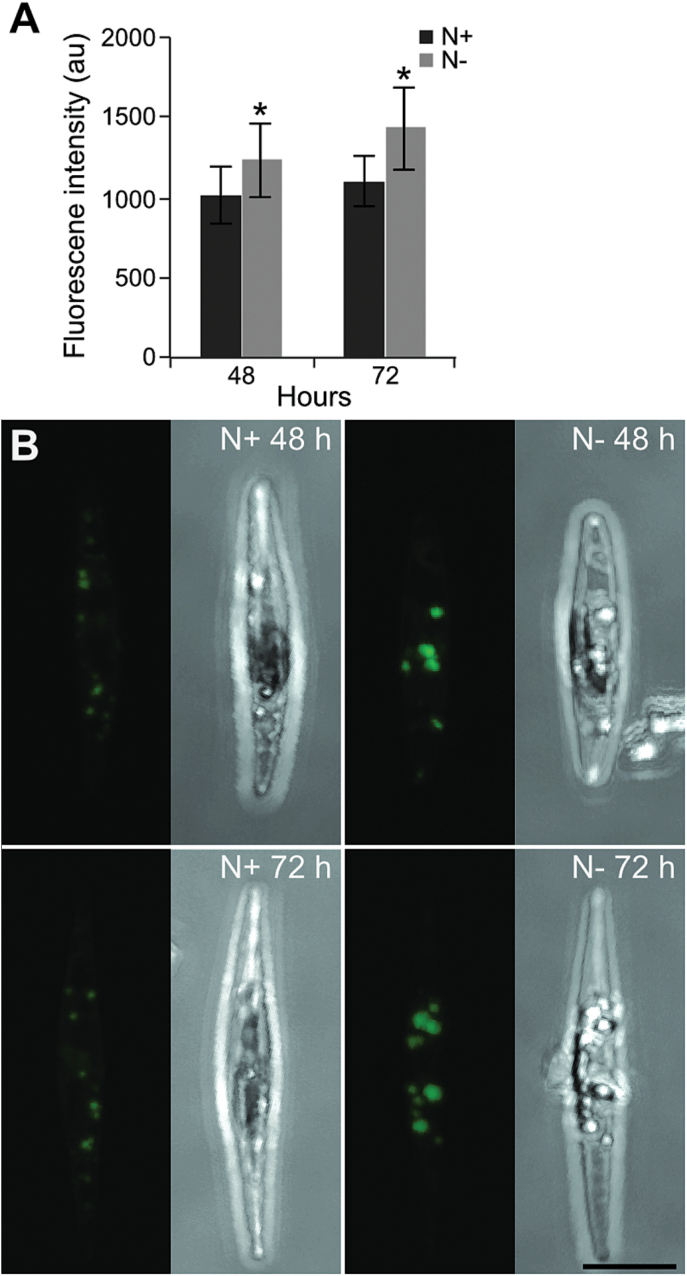
Accumulation of neutral lipids during nitrate deprivation. (A) Fluorescence intensity in *P. tricornutum* cells stained with BODIPY 505/515 at 48 and 72h after N deprivation. The level of lipid fluorescence was measured in 20–30 randomly selected cells using confocal microscopy. Statistical differences (*P<0.01) between nitrate-replete (N+) and nitrate deprived (N–) cultures are indicated. au, Arbitrary units. (B) Z-stack projections of *P. tricornutum* in N+ and N– cultures at 48 and 72h after N deprivation. Bar, 5 μm. (This figure is available in colour at *JXB* online.)

Measurements of chlorophyll *a* and fucoxanthin (the major carotenoid in diatoms) levels per cell showed that the content of these pigments declined progressively in N-deprived cells at both time points, while both pigments were stable in control cells (Table 2). In contrast, the ratio between chlorophyll *a* and fucoxanthin did not change. We also monitored the effect of N deprivation on the activity of PSII. Maximum quantum yield of PSII (F_v_/F_m_), applied as a proxy measure of photosynthesis, was similar in N-replete and N-deprived cultures after 24h. A clear drop in F_v_/F_m_ was observed in N-deprived cells (F_v_/F_m_=0.33) after 72h, while the ratio remained unchanged (F_v_/F_m_=0.68) in the control cultures (Fig. 1B). Quantification of neutral lipids by confocal laser scanning microscopy and BODIPY 505/515 showed that the neutral lipid content increased significantly at 72h (*t*-test: *P*=1.87 E–06) and was 29.3% higher in N-deprived cells compared with N-replete cells (Fig. 2A). Representative images from the analysis showed that the lipid droplets were larger and more strongly stained by the BODIPY marker in N-deprived cells than in replete cells, implying higher lipid accumulation (Fig. 2B).

Metabolite profiling of the responses to N deprivation was performed using GC-MS. Strong effects on the central metabolism were revealed, with a significant decrease in most of the N-containing metabolites and major fatty acids (Supplementary Table S1). The regulation of biosynthesis and significance of distinct metabolites is further discussed in subsequent sections.

### Gene expression

Transcriptome responses at 48 and 72h after N deprivation were analysed using whole-genome oligonucleotide microarrays. The treatment led to strong transcriptome responses: 5279 genes were significantly regulated (*P*<0.05) in N-deprived cultures compared with N-replete cultures 48h after N deprivation. As expected, the stronger N deprivation at 72h affected even more genes (6629). Comparison of the N-replete cultures at 48 and 72h resulted in only 22 significantly regulated genes, probably reflecting higher cell densities (results not shown).

**Fig. 3.**
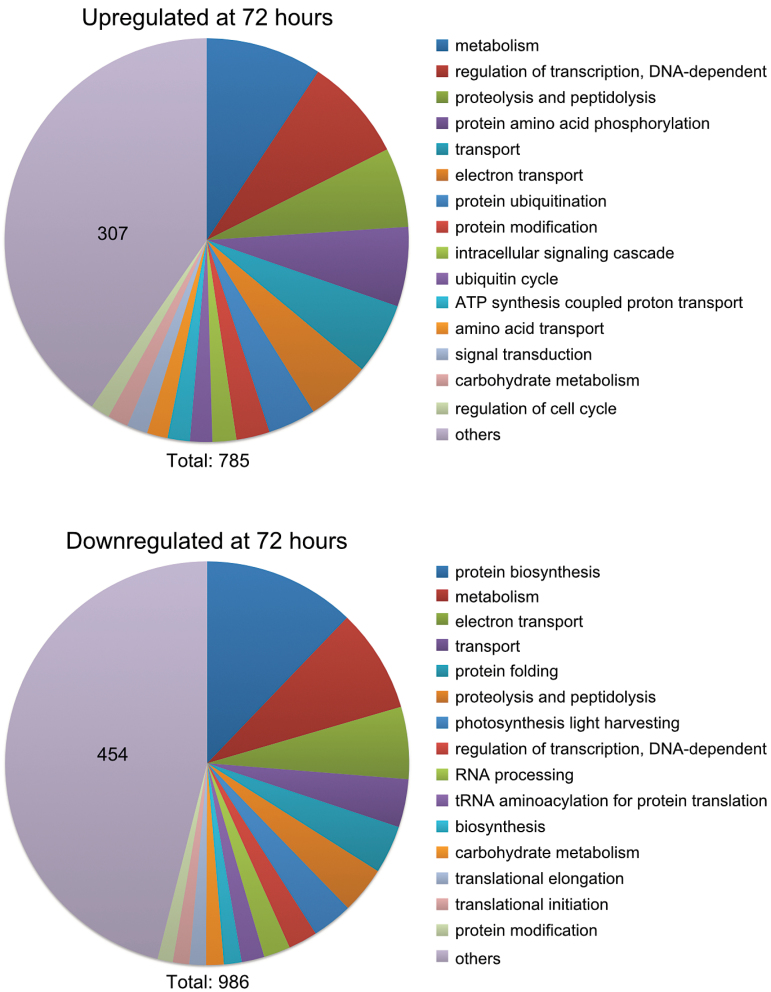
GO analysis of significantly regulated genes after 72h of nitrate deprivation. The dataset was divided into up- and downregulated genes and analysed for process GO terms. The 15 most frequent GO terms are listed, and the rest were combined into ‘others’. The number in the ‘others’ section indicates the number of hits within this category. The total number of GO term hits is listed below the graphs.

GO analysis was performed on the dataset. As the *P. tricornutum* genome is still poorly annotated, GO terms are assigned to a limited number of genes. The GO analysis still provided an overview of the processes most affected by N deprivation. The most enriched GO terms were similar at 48h (Supplementary Fig. S1, available at *JXB* online) and 72h (Fig. 3); however, there were large differences between GO terms enriched in up- and downregulated genes. The most frequent GO term among the downregulated genes was protein biosynthesis. Other GO terms related to ribosomal assembly and translation were also enriched among downregulated genes, indicating reduced protein biosynthesis. Furthermore, photosynthesis light harvesting was the fourth most used GO term among the downregulated genes, indicating downsizing of the light-harvesting apparatus. In contrast, the upregulated genes were enriched in GO terms related to signal transduction and ubiquitination.

**Fig. 4.**
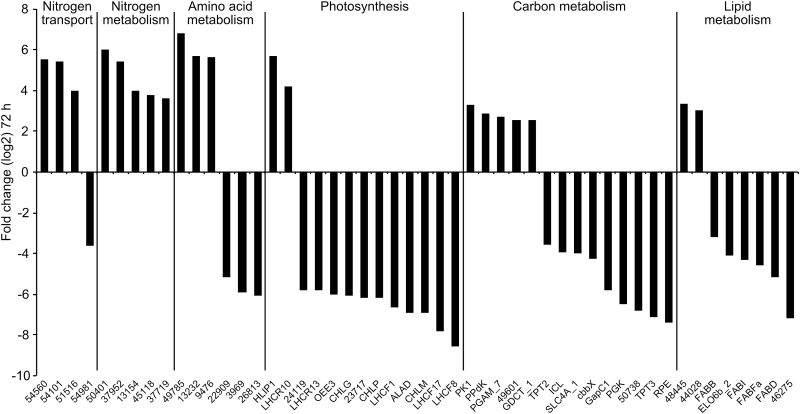
Genes strongly regulated by N deprivation. The genes most up- or downregulated after 72h of nitrate deprivation are shown for the processes listed at the top of the graph. The ratios were log_2_ transformed. Numbers indicate Phatr2 gene IDs.

Strong transcriptional responses were observed for genes encoding proteins involved in processes such as photosynthesis, central carbon metabolism, lipid metabolism, nitrogen metabolism and transport, and amino acid metabolism, as discussed below. The responses of the strongest regulated genes within these categories 72h after N deprivation are shown in Fig. 4. In order to verify the results of microarray analysis, qRT-PCR was performed on five selected genes involved in photosynthesis, and N and carbon metabolism, respectively, that were differentially regulated at 48 and 72h in the microarray analysis. The qRT-PCR results correlated well with the microarray analysis (Supplementary Fig. S2, available at *JXB* online).

## Effect of N deprivation on N metabolism

### N uptake and assimilation

**Fig. 5.**
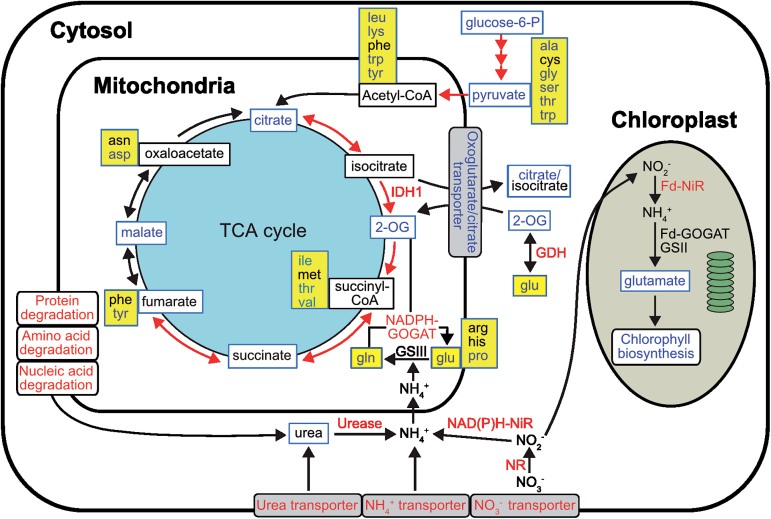
Cellular pathways and processes related to N metabolism under N deprivation in *P. tricornutum*. Metabolites detected are indicated by a blue box frame. Red, blue, and black text indicate up-, down-, and no regulation of pathways, genes, or metabolites by N deprivation, respectively. Amino acids are indicated by a yellow background. Red arrows depict gene transcripts found to be upregulated. Fd-GOGAT, ferredoxin-dependent glutamate synthase; GSII, ferredoxin-dependent glutamine synthetase; Fd-NiR, ferredoxin-dependent nitrite reductase; GDH, glutamate dehydrogenase; GSIII, bacterial-origin glutamine synthetase; IDH, isocitrate dehydrogenase; NADPH-GOGAT, NAD(P)H-dependent glutamate synthase; NAD(P)H-NiR, NAD(P)H-dependent nitrite reductase; NR, nitrate reductase.

Transcriptional responses to N deprivation of *P. tricornutum* showed that uptake, assimilation, and scavenging mechanisms were activated (Fig. 5). In our experiment, transcript levels of genes involved in $NO_{3}^{–}$ , $NH_{4}^{+}$ , and urea transport were upregulated (Fig. 4 and Supplementary Dataset S1, available at *JXB* online). Of four ammonium transporters detected in our microarray data, three were upregulated. The induction of a nitrate transporter (Phatr2_54101) was confirmed by qRT-PCR (Supplementary Fig. S2). Increased transcription of genes encoding nitrate reductase (NR) and both NAD(P)H- and Fd-dependent nitrite reductase was observed at 72h after deprivation (Figs 4 and 5). Interestingly, two genes encoding molybdopterin biosynthesis proteins were induced (Supplementary Dataset S1). These enzymes might be orthologues of the *Arabidopsis thaliana* cofactor of NR and xanthine dehydrogenase CNX5 and CNX2, respectively (Schwarz and Mendel, 2006). The biosynthesis of molybdenum cofactor (Moco), which forms the active site of molybdenum (Mo) enzymes in eukaryotes, involves six enzymes. The qRT-PCR result also confirmed upregulation of the CNX5 orthologue (Phatr2_34373; Supplementary Fig. S2). None of the genes encoding plastidial GSII/Fd-GOGAT and mitochondrial GSIII (GLNA), which are required for ammonium assimilation, were regulated (*P*<0.05). However, increased transcript levels of two different isoforms of NAD(P)H-dependent glutamate synthase (NADPH-GOGAT, *GltD* and *GltX*) were observed in N-deprived cells (Fig. 5). Glutamate dehydrogenase (GDH) is another enzyme that catalyses the reversible conversion of 2-oxoglutarate (2-OG) to glutamate. We observed increased expression of an NADP-GDH (Phatr2_13951; Fig. 5 and Supplementary Dataset S1).

#### N scavenging from various organic compounds

We observed induction of genes encoding two glutamyl-tRNA(Gln) amidotransferase-like proteins (Phatr2_50401 and Phatr2_45118) and three acetamidase/formamidases (Phatr2_54476, Phatr2_37952, and Phatr2_37719) at both time points in N-deprived cells (Fig. 4). Phylogenetic analyses indicated that Phatr2_54476 is related to FmdA-type formamidases (Supplementary Fig. S3, available at *JXB* online); the main substrate of *Methylophilus methylotropus* FmdA (Wyborn *et al.*, 1994), as well as lupin LaFmd (Rath *et al.*, 2010), is formamide. Phatr2_37952 and Phatr2_37719, which were strongly induced at both time points, encode amidohydrolases belonging to a poorly characterized clade with low similarity to FmdA-type formamidases (Supplementary Fig. S3).

#### Purine and pyrimidine biosynthesis and degradation

Most of the transcripts involved in biosynthesis of purine and pyrimidine were downregulated (Supplementary Dataset S1). Simultaneously, we observed upregulation of several transcripts involved in their catabolic processes, such as purine and pyrimidine deaminases. Furthermore, uracil-xanthine permease (Phatr2_16991) and adenine/guanine permease (Phatr2_17344) transcripts were upregulated in N-deprived cultures; the response of the latter was confirmed by qRT-PCR analysis (Supplementary Fig. S2). Urease (Phatr2_29702), which catalyses the hydrolysis of urea into CO_2_ and $NH_{4}^{+}$ , was also transcriptionally induced following N deprivation (Supplementary Dataset S1).

#### Protein biosynthesis, folding, and degradation

N deprivation influenced both biosynthesis and degradation of proteins and amino acids. Most amino acid biosynthesis pathways were transcriptionally repressed; the strongest downregulation was found for transcripts encoding homoserine dehydrogenase (Phatr2_26813) and *N*-acetylglutamate kinase (Phatr2_3969) (Fig. 4). Reduction of protein biosynthesis could be observed as a decrease in mRNA levels of many genes encoding aminoacyl-tRNA synthetases, as well as ribosomal subunits and translation elongation factors. The transcription levels of genes encoding 18 peptidylprolyl isomerases and two protein disulfide isomerases that catalyse protein folding were also reduced. In contrast, the transcript levels of genes encoding amino acid degradation enzymes, such as those related to catabolism of branched-chain amino acids, were upregulated (Fig. 4). Five identified autophagy-related genes were upregulated at one or both time points in N-deprived cells (Supplementary Dataset S1). The mRNA levels of several genes involved in ubiquitination were upregulated, but most of the proteasome subcomponents were downregulated.

#### Effect of N deprivation on photosynthesis and pigment biosynthesis

Glutamate, the main precursor of chlorophyll biosynthesis, declined under N-deprived conditions (Supplementary Table S1). In line with the reduced chlorophyll *a* and fucoxanthin levels in N-deprived cells, the expression levels of most of the genes encoding enzymes involved in the chlorophyll *a* and carotenoid biosynthetic pathways were also repressed (Figs 4 and 5, and Supplementary Dataset S1). Of 39 differentially regulated genes encoding LHC proteins, only red algal-like *LHCR10* and two high-light-induced proteins (*HLIP1* and *HLIP1b*) were significantly upregulated at both time points, whereas the LI818-like *LHCX4* and *LHCR7* showed moderate upregulation 72h after N deprivation (Fig. 4). Among the downregulated LHCs, repression of *LHCF1* was confirmed by qRT-PCR (Supplementary Fig. S2). Similarly, a majority of the transcripts involved in photosynthesis were downregulated (Figs 4 and 5, and Supplementary Dataset S1). Furthermore, a chloroplastic ferredoxin-NADP reductase (Phatr2_23717) was strongly downregulated, indicating that NAD(P)H production through photosynthesis was reduced (Fig. 4).

### Effect of N deprivation on carbon and lipid metabolism

#### Carbon fixation

**Fig. 6.**
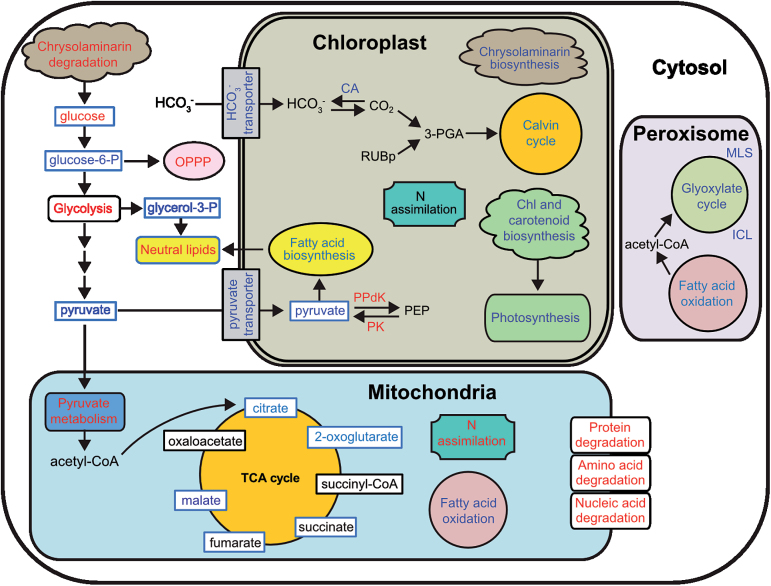
Cellular pathways and processes affected under N deprivation in *P. tricornutum*. Metabolites detected are indicated by a blue box frame. Red, blue,and black text indicates up-, down-, and no regulation of pathways, genes, or metabolites by N deprivation, respectively. 3-PGA, 3-phosphoglycerate; CA, carbonic anhydrase; ICL, isocitrate lyase; MLS, malate synthase; OPPP, oxidative pentose phosphate pathway; PEP, phosphoenolpyruvate; PK, pyruvate kinase; PPdK, pyruvate orthophosphate dikinase; RuBP, ribulose-1,5-bisphosphate.

Downregulation of several genes connected to the biophysical carbon-concentrating mechanism was observed in N-deprived cells. Of five carbonic anhydrase (CA) genes related to the biophysical carbon-concentrating mechanism that were significantly regulated 72h after N deprivation (Supplementary Dataset S1), transcript levels of *CA-III* and two β-CAs (*PtCa1* and *PtCa2*) decreased. A chloroplast bicarbonate transporter (*SLC4A_1*) was also repressed. In accordance with the β-CA and SLC4A_1 downregulation, a majority of the genes encoding enzymes of the Calvin cycle were downregulated (Figs 4 and 6). Upregulation of several transcripts encoding enzymes involved in the mitochondrial decarboxylation under N-deprived conditions was observed in N-deprived cells (Supplementary Dataset S1). Downregulation of plastid-localized pyruvate carboxylase 2 (*PYC2*) was confirmed by qRT-PCR analysis (Supplementary Fig. S2).

#### TCA cycle

Consistent with upregulation of the mitochondrial decarboxylation, TCA cycle transcripts were induced (Figs 5 and 6, and Supplementary Dataset S1). However, genes encoding enzymes towards the end of the TCA cycle were not regulated. Aconitate hydratase (Phatr2_26290) and isocitrate dehydrogenase (Phatr2_14762) transcripts showed the highest level of upregulation. In contrast to the upregulation of TCA transcripts, we observed a decrease in the levels of most of the metabolite intermediates of the TCA cycle (Supplementary Table S1).

#### Chrysolaminarin biosynthesis and degradation

We observed repression of several genes encoding enzymes potentially involved in gluconeogenesis, as well as chrysolaminarin biosynthesis (Kroth *et al.*, 2008; Chauton *et al.*, 2013), especially 72h after N deprivation (Fig. 6). Inversely, transcript levels of genes encoding enzymes for chrysolaminarin degradation, such as exo-1,3-β-glucosidases, increased. Chrysolaminarin degradation produces glucose; indeed, glucose levels were higher in N-deprived cells (Fig. 6 and Supplementary Table S1). Consistent with the increased glucose level, cytosolic glucokinase was also induced in our experiment.

#### Oxidative pentose phosphate pathway (OPPP), glycolysis, and pyruvate metabolism

Surprisingly, all OPPP transcripts were induced (Fig. 6). Transcripts of most of the putatively cytosolic glycolytic enzymes, such as phosphoglycerate mutase (PGAM_7), increased in our experiment, while transcript levels of several plastidial enzymes, such as glyceraldehyde-3-phosphate dehydrogenase (GAPC1), showed the opposite regulation (Fig. 4). In contrast to the transcriptional induction of glycolytic enzymes, metabolite levels of glucose-6-phosphate, fructose-6-phosphate, and pyruvate declined (Supplementary Table S1), which might be the result of their quick conversion to other metabolites. Consistent with the decrease in pyruvate, transcript levels of several genes responsible for pyruvate metabolism were upregulated.

#### Fatty acid biosynthesis and degradation

Most transcripts related to the chloroplast fatty acid biosynthetic pathway were strongly downregulated (Fig. 4 and Supplementary Dataset S1, available at *JXB* online); the only upregulated transcript was 3-oxoacyl-[acyl-carrier-protein] synthase (FABFb) (Phatr2_18940). We also observed lower levels of total free fatty acids in N-deprived cells (Supplementary Table S1), which might be a consequence of their incorporation into TAG.

#### Membrane lipid remodelling and TAG biosynthesis

**Fig. 7.**
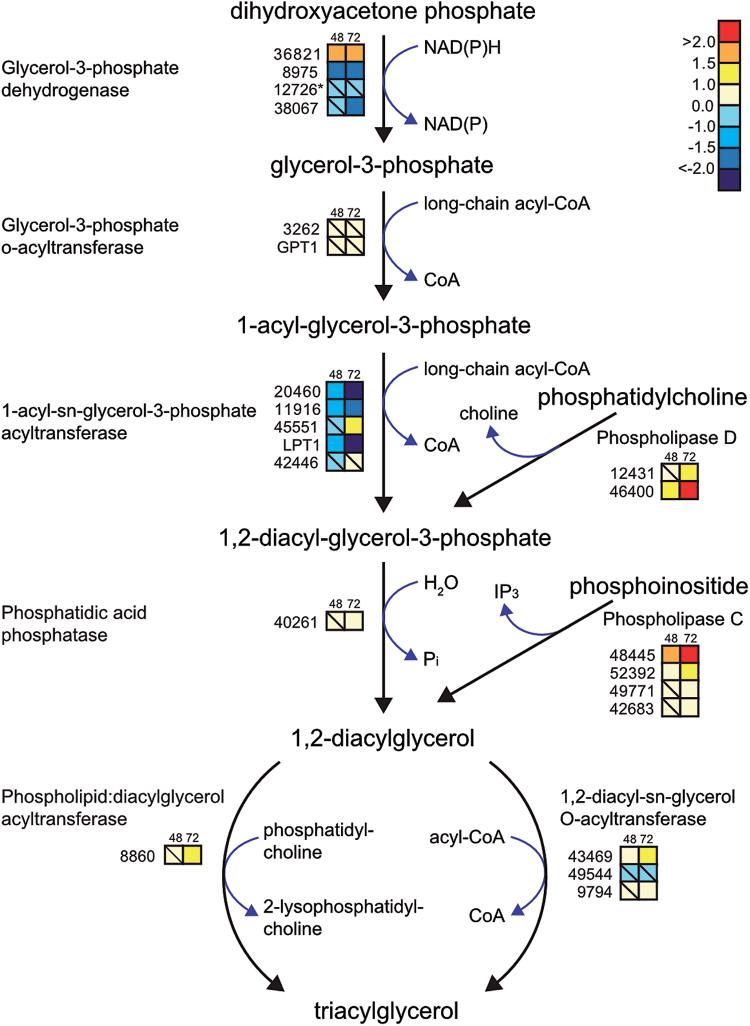
Transcriptional changes in genes related to TAG biosynthesis in response to N deprivation. Coloured squares indicate the regulation pattern of genes encoding putative enzymes functioning in the TAG biosynthetic pathway after 48 and 72h of N deprivation, compared with N-replete cultures. Squares with a diagonal line inside indicate non-significant regulation (*P*>0.05). The scale on the right represents gene expression ratio values, which were log_2_ transformed. Numbers indicate Phatr2 gene IDs. Gene ID 12726 (marked with an asterisk) belongs to the Phatr1 database (http://genome.jgi-psf.org/Phatr1/Phatr1.home.html).

TAG biosynthetic pathways are illustrated in Fig. 7. Of three differentially regulated glycerol-3-phosphate dehydrogenases, transcript levels of Phatr2_36821 increased in N-deprived cells (Fig. 7). This enzyme consumes NAD(P)H to convert dihydroxyacetone phosphate, an intermediate in glycolysis, to glycerol-3-phosphate. Subsequent transfer of two acyl-CoAs to glycerol-3-phosphate by glycerol-3-phosphate acyltransferase and acyl-glycerol-3-phosphate acyltransferase (AGPAT) result in the formation of phosphatidic acid. Of the five detected isoforms of AGPAT, only one putative isoform (Phatr2_45551) displayed increased transcript levels 72h after deprivation, whereas three others (Phatr2_20460, Phatr2_11916, and LPT1) were suppressed. Phosphatidic acid is dephosphorylated to diacylglycerol, the main precursor of TAG. This process is catalysed by phosphatidic acid phosphatase; a putative PAP (Phatr2_40261) was weakly upregulated. Incorporation of the third fatty acyl-CoA into glycerol-3-phosphate backbone by diacylglycerol *O*-acyltransferase (DGAT) completes TAG formation. The mRNA levels of two isoforms of DGAT (Phatr2_43469 and Phatr2_9794) were induced in our experiment. Although we could detect TAG accumulation at both the molecular and physiological level, the transcript abundance of several TAG lipases was induced in N-deprived cells.

Upregulation of four isoforms of phospholipase C and two isoforms of phospholipase D indicated that membrane phospholipids are degraded under N deprivation to provide the TAG precursors phosphatidic acid and diacylglycerol (Fig. 7). The highest induction was seen for phospholipase C (Phatr2_48445), with an approximately 4- and 10-fold increase 48 and 72h after deprivation, respectively. Transcript levels of a putative phospholipid:diacylglycerol acyltransferase enzyme (PDAT, Phatr2_8860) were also upregulated at 72h in N-deprived cells (Fig. 7).

## Discussion

Previous studies in diatoms and other microalgae have demonstrated that these organisms undergo dramatic metabolic changes in response to N starvation (Hockin *et al.*, 2012; Valenzuela *et al.*, 2012; Yang *et al.*, 2013). Performing an integrated analysis of the response to N deprivation in *P. tricornutum*, we confirmed these modifications at the physiological, metabolite, and transcriptome levels.

### Reprogramming of N metabolism

We observed a higher C:N ratio than that suggested by the Redfield ratio (Redfield, 1934) in N-replete and N-deprived cultures, indicating that the C:N:P composition of phytoplankton and marine particulate matter is flexible, especially in nutrient-deprived cells (Geider and La Roche, 2002). Furthermore, an increase in the C:N ratio of N-deprived cultures is probably a result of biomass increase after N exhaustion.

Although transcriptional regulation is indicative and not necessarily directly linked to changes at protein level, some trends are evident. Due to a decrease in the N content of N-deprived cells, many processes connected to N metabolism were affected. A major response to N deprivation was to increase the cellular capacity for N uptake and nitrate reduction to ammonium, as observed at the transcript level. This phenomenon was reported previously in N-deprived *P. tricornutum* and other microalgae at the transcriptome level (Mock *et al.*, 2008; Miller *et al.*, 2010; Valenzuela *et al.*, 2012) (Fig. 7). However, the downregulation of one ammonium transporter (Phatr2_54981) was in agreement with previous reports (Haimovich-Dayan *et al.*, 2013; Krell *et al.*, 2007; Yang *et al.*, 2013). The induction of two molybdopterin biosynthesis genes could be related to an increased need of Moco for NR and xanthine dehydrogenase in *P. tricornutum*. In contrast to our results, a *NR* gene was repressed under N stress in *Emiliania huxleyi*; the authors postulated that expression of the *NR* gene is stimulated under high $NO_{3}^{–}$ concentrations (Bruhn *et al.*, 2010). In addition, they observed no co-ordination between the regulation of nitrate reduction at the transcript and protein levels. Despite the increase in the expression level of genes encoding nitrate-reducing enzymes in N-deficient *T. pseudonana* (Mock *et al.*, 2008), their protein levels were found to decrease in a proteomic study (Hockin *et al.*, 2012); the authors postulated that the levels of these enzymes might be controlled by post-transcriptional modifications. In photosynthetic eukaryotes, ammonium assimilation primarily occurs inside the chloroplast (Bowler *et al.*, 2010). Although we could not detect any regulation of *GSII/Fd-GOGAT* or mitochondrial *GLNA*, increased transcript levels of two different NADPH-GOGAT isoforms (*GltD* and *GltX*) indicated that cells increased their N scavenging mechanisms to assimilate more ammonium from other pathways such as protein and amino acid degradation (Fig. 5). In addition, the upregulated levels in N-deprived cells of another ammonium assimilating enzyme, NADP-GDH, might also contribute to ammonium assimilation. GDH is generally believed to act as a catabolic enzyme, catalysing the oxidative deamination of glutamate to 2-OG, and previous studies have implied that GDH is a minor contributor to $NH_{4}^{+}\;$ assimilation (Zehr and Falkowski, 1988; Guerra et al., 2013). However, the observed decrease in 2-OG levels might indicate that GDH also can perform the anabolic reaction to scavenge $NH_{4}^{+}\;$ under N deprivation. Previous studies have demonstrated that diatoms are able to use other sources of N, such as amides, amines, urea, and amino acids (Shah and Syrett, 1982; Baker *et al.*, 2009). Besides the increase in N-scavenging mechanisms, upregulation of amidases and acetamidase/formamidases indicates that *P. tricornutum* can degrade organic N sources such as amides and formamide from intracellular or possible extracellular sources to produce ammonium when faced with N deprivation, as observed in N-deficient *Aureococcus anophagefferens* (Wurch *et al.*, 2011). In support of our results, a formamidase transcript was induced under N stress in *E. huxleyi* (Bruhn *et al.*, 2010).

Biosynthesis of several amino acids relies on the availability of glutamate, which declined after N deprivation as a consequence of a reduced N pool (Supplementary Table S1). The lower glutamate level compared with the control resulted in a strong drop in levels of other amino acids and repression of amino acid biosynthetic pathways. A decline in the cellular amino acid pool as a result of N deprivation is consistent with results from other diatoms (Granum *et al.*, 2002). Simultaneous with the suppression of amino acid biosynthesis, increased degradation of amino acids through various catabolic pathways was observed at the transcript level, which produced several carbon-containing intermediates that can enter the TCA cycle. Consistent with increased transcripts associated with catabolism of branched-chain amino acids in our experiment, Ge *et al.* (2014) suggested that branched-chain amino acid degradation directs carbon and energy towards TAG accumulation in N-deprived *P. tricornutum*.

All purine and pyrimidine nitrogen compounds originate from amino acids (glutamine, aspartate, and glycine) (Zrenner *et al.*, 2006). Reduced biosynthesis of purine and pyrimidines is probably a result of decreases in the amino acid pools (Supplementary Table S1) and cell growth (Fig. 1A). At the same time, recycling of purines and pyrimidines provides the cells with an important N source during N deprivation. Upregulation of the urease gene could also be related to purine degradation. $NH_{4}^{+}\;$ produced from the hydrolysis of urea is hypothesized to be redirected to mitochondria for amino acid biosynthesis via mitochondrial GS/GOGAT (Allen *et al.*, 2011). The strong induction of purine/pyrimidine permeases might explain the ability of diatoms to import purine and pyrimidine from the environment under limited N conditions, as reported by Allison and Syrett (1987). However, Berg *et al.* (2008) showed that the purine permease *AaURA* is expressed during growth of A. anophagefferens on a number of N sources, indicating its role as an important nitrogen source for proliferation of this organism. The high expression of *AaURA* might be related to the growth habitat of *A. anophagefferens* in shallow coastal waters, which are in close contact with sediments rich in dissolved organic N.

Eukaryotes utilize autophagy and the ubiquitin–proteasome system for protein degradation (Onodera and Ohsumi, 2004). The ubiquitin–proteasome system is used for rapid degradation of proteins and acts mainly to degrade short-lived proteins such as transcription factors, while the turnover of long-lived proteins, which constitute 99% of cellular proteins, is processed by autophagy (Onodera and Ohsumi, 2004). While N-limited *A. anophagefferens* showed moderate downregulation of two autophagy-related genes (Berg *et al.*, 2008), the transcript levels of several autophagy-related genes in our experiment and during N limitation of the green microalga *Neochloris oleoabundans* (Rismani-Yazdi *et al.*, 2012) were induced. Although autophagy generally is not a selective protein degradation process, selective autophagy was stimulated under conditions of nutritional stress, especially N deficiency, in yeast and plants (Onodera and Ohsumi, 2004; Yoshimoto *et al.*, 2004). Therefore, the induction of autophagy-related genes can be explained as a response to N deprivation by selectively degrading excessive proteins into amino acids that are recycled to protein biosynthetic pathways in order to maintain cellular homeostasis. Autophagy is highly regulated at the protein level (Klionsky and Emr, 2000); therefore, it would be worthwhile looking at regulation of autophagy components at the protein level to better understand their role under N deprivation.

Chlorosis is one of the main responses of diatoms to N deprivation. The reduced chlorophyll *a* level in N-deprived cells (Table 2) is probably caused at least partly by repression of its biosynthesis. In addition, the co-ordinated decrease in chlorophyll *a* and fucoxanthin content under N deprivation suggests that the biosynthesis of these two pigments is co-ordinated under N deprivation; a similar result was also observed in a previous study in *P. tricornutum* (Geider *et al.*, 1993). In contrast, N limitation in *Chaetoceros gracilis* led to changes in the chlorophyll *a*:fucoxanthin ratio (Cleveland and Perry, 1987). The decrease in pigment biosynthesis and LHC proteins corresponds to a reduced photosynthetic apparatus of N-deprived cells and a lower requirement for pigments. These results clearly indicate that N deprivation reduces the photosynthetic efficiency, in agreement with the observed reduction in maximum quantum yield of PSII (F_v_/F_m_) (Fig. 1B). Despite the downregulation of LHCF proteins, induction of LHCXs was reported under several stresses in diatoms (Zhu and Green, 2008). Induction of *LHCR10* expression by N deprivation was also observed by Yang *et al.* (2013); furthermore, both LHCX and LHCR-II genes, to which *LHCR7* and *LHCR10* belong, are induced by high light (Nymark *et al.*, 2009, 2013). The increased transcript levels of *LHCX4*, along with two LHCRs and two HLIPs, may be related to a photoprotective role during acclimation to low N levels. In summary, N-deprived *P. tricornutum* modified N metabolism in order to reduce synthesis of nitrogenous compounds and catabolize excessive N-containing compounds in favour of essential N compounds.

### Remodelling of carbon metabolism

The major pathway used by diatoms for carbon fixation is the Calvin cycle. Since β-CAs and $HCO_{3}^{–}$ transporters are required to concentrate inorganic carbon in the vicinity of Rubisco, their downregulation repressed a majority of the genes encoding enzymes of the Calvin cycle (Fig. 6). Although the Calvin cycle was downregulated, cells might employ other mechanisms such as pyruvate orthophosphate dikinase to dissipate excess energy around the photosystems to reduce the production of reactive oxygen species under N deficiency, as reported by Haimovich-Dayan *et al.* (2013).

Upregulation of several transcripts encoding enzymes involved in the mitochondrial decarboxylation under N-deprived conditions leads to production of oxaloacetate and pyruvate (Supplementary Dataset S1). Oxaloacetate can replenish C_4_ acids of the TCA cycle, whereas pyruvate can enter the TCA cycle or fatty acid biosynthesis. The TCA cycle could also be upregulated in response to high levels of protein and amino acid degradation, which generates TCA cycle intermediates and provides precursors for resynthesis of certain amino acids, as observed by Hockin *et al.* (2012). Furthermore, strong upregulation of the genes encoding aconitate hydratase and isocitrate dehydrogenase leads to production of 2-OG, which acts as a precursor in ammonium assimilation. Malate from the TCA cycle could also be directed to the fatty acid biosynthetic pathway through NADP-dependent malic enzyme (Supplementary Dataset S1). Similar regulation of the TCA cycle was reported for other diatoms under N deprivation (Bender *et al.*, 2014). Thus, a co-ordinated upregulation of the TCA cycle and mitochondrial decarboxylation might shift the flow of carbon skeletons towards fatty acid biosynthesis.

Degradation of chrysolaminarin releases glucose. Glucose cannot enter the metabolic pathway directly and must be converted to glucose-6-phosphate by cytosolic glucokinase. Phosphorylated glucose could further enter the glycolytic pathway and/or OPPP (Fig. 6). Upregulation of OPPP produces NAD(P)H supplying NAD(P)H-dependent pathways like lipid synthesis and nitrogen assimilation. Utilization of glucose-6-phosphate through glycolysis produces energy in the form of ATP and NAD(P)H, as well as the glycolysis end product, pyruvate. Further metabolism of pyruvate produces acetyl-CoA, which can enter the TCA cycle, or *de novo* fatty acid biosynthesis in the chloroplast. Thus, increased degradation of chrysolaminarin, along with induction of OPPP and glycolysis, could provide N-deprived cells with reducing equivalents to balance reduced NAD(P)H production from photosynthesis, as well as carbon fluxes for the TCA cycle and fatty acid biosynthesis.

The microarray data showed downregulation of *de novo* fatty acid biosynthesis genes (Fig. 4 and Supplementary Data S1), supporting the observed decrease in free fatty acids (Supplementary Table S1). In contrast, Yang *et al.* (2013) reported increased total fatty acid levels in N-deprived cells, while RNA-sequencing data from their experiment showed that most of the transcripts involved in chloroplast *de novo* synthesis of fatty acids were downregulated. We conclude that, under exponential growth, the high cell division rate leads to a high demand for membrane lipids in newly synthesized cells. In contrast, cell division slows or halts in cells faced with N limitation, and there is less need for membrane lipids and fatty acids. Even though *de novo* synthesis of fatty acids is downregulated, fatty acids are still produced and accumulate in the form of neutral lipids. Moreover, repression of the fatty acid β-oxidation pathway might direct fatty acids from degradation of membrane lipids to TAG production.

Under favourable conditions, fatty acids are incorporated into membrane lipids. The main classes of lipids in diatom chloroplasts are sulphoquinovosyldiacylglycerol, monogalactosyldiacylglycerol, digalactosyldiacylglycerol, phosphatidylglycerol, and phosphatidylcholine; however, phosphoglyceride levels are higher in the endoplasmic and plasma membranes (Hu *et al.*, 2008; Goss and Wilhelm, 2010). Therefore, the downregulation in membrane lipid biosynthesis could be related to a reduced demand for membrane lipids due to the reduced cell growth rate. A major effect of N starvation in many microalgae is the switch in lipid production towards neutral lipid accumulation, mainly TAGs, which act primarily as energy reserves inside the cells (Hu *et al.*, 2008). Increased levels of neutral lipids were also observed in our study (Fig. 2). TAG can be synthesized through different pathways, one of which uses glycerol-3-phosphate as a precursor (Fig. 7) (Hu *et al.*, 2008). The accumulation of TAG was consistent with a decrease in the amount of free glycerol-3-phosphate inside the cells (Supplementary Table S1), implying that this compound might be used as a precursor for TAG biosynthesis. Furthermore, TAG can also accumulate via degradation of glycerophospholipids. The increased expression of phospholipase C and phospholipase D suggests that these enzymes contribute substantially to TAG accumulation through processing of phospholipids. An acyl-CoA-independent mechanism for biosynthesis of TAG from diacylglycerol using phospholipids as acyl donors is catalysed by a putative gene encoding phospholipid:diacylglycerol acyltransferase (PDAT, Phatr2_8860). This could also contribute to TAG accumulation upon N deprivation. The upregulation of several TAG lipases could be related to saturated lipid bodies and could act to recycle previously synthesized TAGs with new TAGs, or they might have been induced to make modifications in TAG structure. Overall, our results suggest that both *de novo* TAG biosynthesis and remodelling of membrane lipids play important roles in TAG accumulation under N deprivation.

### Conclusions

A combined analysis of transcriptional and non-targeted metabolite profiling, along with physiological and biochemical experiments, revealed transcriptional, metabolic, and physiological acclimation in the diatom *P. tricornutum* under conditions of N deprivation. The global expression data suggested that *P. tricornutum* is able to remobilize N through catabolism of internal N-containing resources such as amino acids and proteins. N deprivation was also accompanied by a reduction of pigment pools and photosynthetic capacity. We also showed large changes in genes related to carbon and lipid metabolism. Decreased levels of carbon skeletons due to suppression of the Calvin cycle were compensated by breakdown of chrysolaminarin, leading to upregulation of OPPP, cytosolic glycolysis, pyruvate metabolism, and the TCA cycle. These pathways provide precursors for fatty acid biosynthesis. In addition, remodelling of membrane lipids and upregulation of the *de novo* TAG biosynthetic pathway was further supported by increased levels of neutral lipids, indicating TAG accumulation under N deprivation. Our study provides a detailed picture of *P. tricornutum* acclimation to N deprivation, and can be used as a guide for future metabolic manipulations to increase TAG production.

## Supplementary data

Supplementary data are available at *JXB* online.

Supplementary Fig. S1. GO analysis of significantly regulated genes after 48h of nitrate deprivation.

Supplementary Fig. S2. qRT-PCR analysis of selected genes.

Supplementary Fig. S3. Phylogenetic analysis of FmdA-type amidases/formamidases in *P. tricornutum*.

Supplementary Table S1. Tentatively identified algal metabolites based on GC-MS profiling.

Supplementary Table S2. Genes analysed by qRT-PCR and their respective primers.

Supplementary Dataset S1. Representative mRNA transcripts grouped by cellular pathway.

Supplementary Data

## Abbreviations:

CA: carbonic anhydrase
CCM: carbon-concentrating mechanism
GC-MS: gas chromatography2-OG, 2-oxoglutarate; /mass spectrometry
GDH: glutamate dehydrogenase
GO: Gene Ontology
HPLC: high-performance liquid chromatography
LHC: light-harvesting complex
NR: nitrate reductase
N: nitrogen
OPPP: oxidative pentose phosphate pathway
PSII: photosystem II
qRT-PCR: quantitative real-time PCR
TAG: triacylglycerol
TCA: tricarboxylic acid.
