# Supplementary material for: Whole-cell response to nitrogen deprivation in the diatom Phaeodactylum tricornutum
Source: J Exp Bot. 2015 Jul 10;66(20):6281–96. doi: 10.1093/jxb/erv340 (PMC4588885; doi:10.1093/jxb/erv340)
Supplement: Supplementary Data [file supp_66_20_6281__index.html]

Whole-cell response to nitrogen deprivation in the diatom Phaeodactylum tricornutum — Whole-cell response to nitrogen deprivation in the diatom Phaeodactylum tricornutum — Whole-cell response to nitrogen deprivation in the diatom Phaeodactylum tricornutum — Supplementary Data 

# Whole-cell response to nitrogen deprivation in the diatom *Phaeodactylum tricornutum*

## Supplementary Data

Data files

- Supplementary Data - Supplementary Data
- Supplementary Data - Supplementary Data
