# Supplementary material for: Whole-cell response to nitrogen deprivation in the diatom Phaeodactylum tricornutum
Source: J Exp Bot. 2015 Jul 10;66(20):6281–96. doi: 10.1093/jxb/erv340 (PMC4588885; doi:10.1093/jxb/erv340)
Supplement: Supplementary Data [file supp_erv340_jexbot140517_file001.pdf]

Whole cell response to nitrogen depletion in the diatom *Phaeodactylum tricornutum*

Leila Alipanah, Jens Rohloff, Per Winge, Atle M. Bones and Tore Brembu

Supplementary Data

Figs S1-S3
Table S1

Supplementary Fig. S1

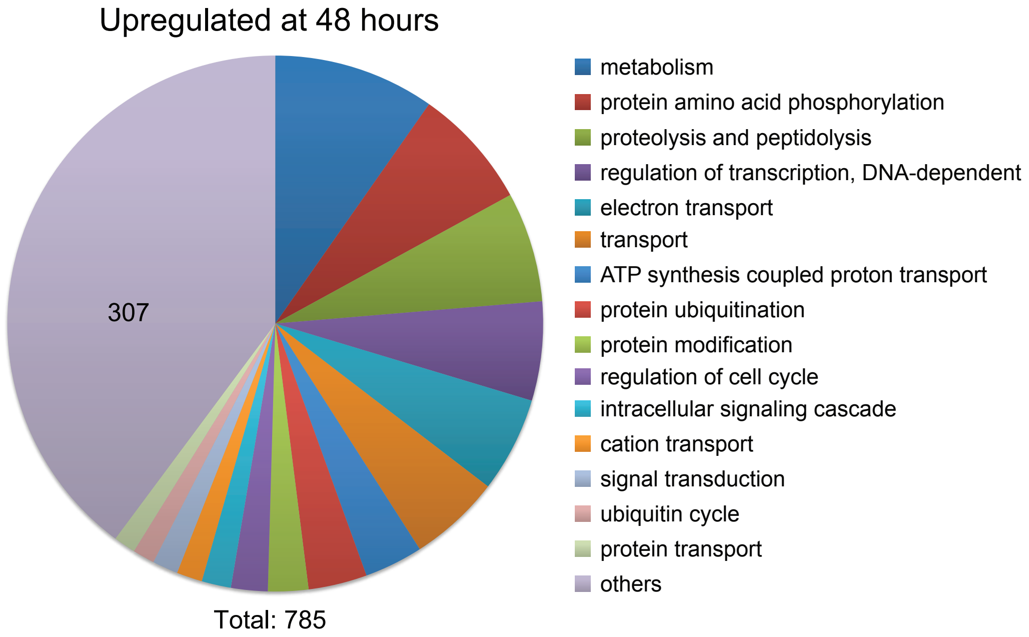

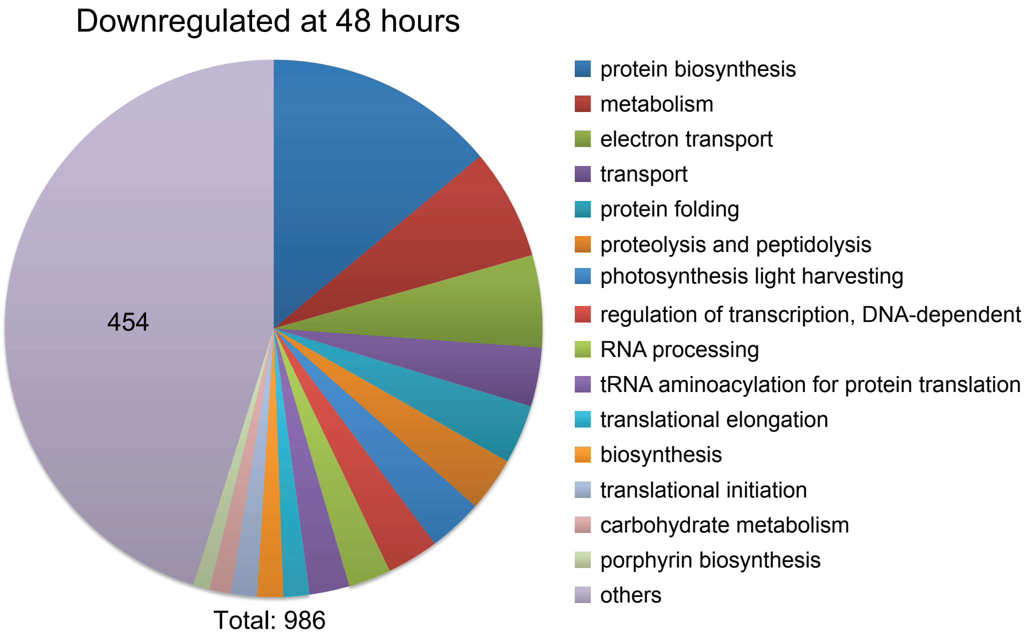

Fig. S1. GO analysis of significantly regulated genes after 48 h of nitrate deprivation. The dataset was divided into up- and down-regulated genes and analyzed for process GO terms. The 15 most frequent GO terms were listed, and the rest were combined into "others". The number in the "others" section indicates the number of hits within this category. The total number of GO term hits is listed below the diagram.

Supplementary Fig. S2

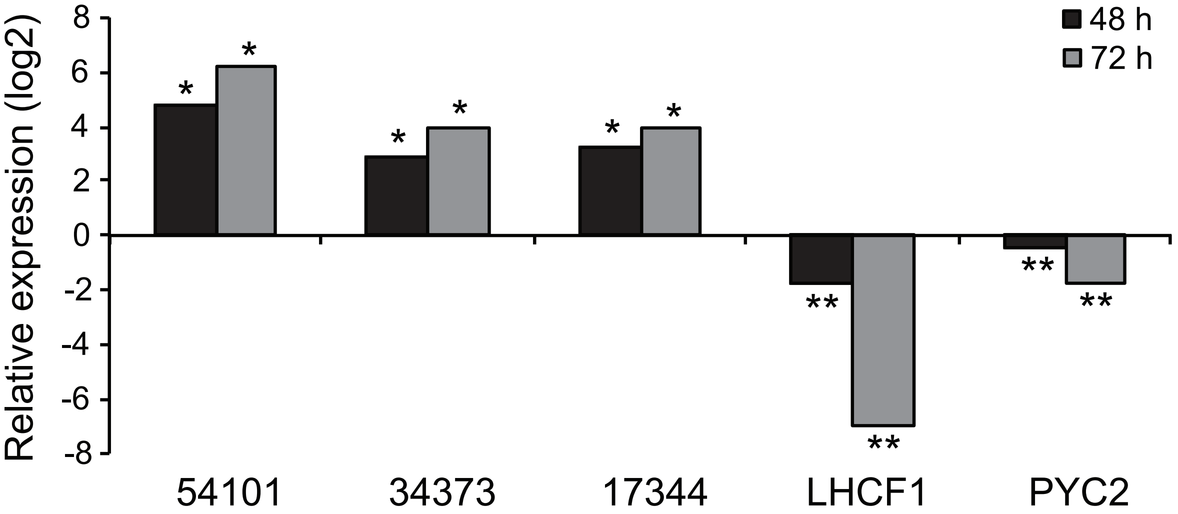

Fig. S2. qRT-PCR analysis of gene responses to nitrate deprivation in *P. tricornutum*. Bars represent relative gene expression values (log2 transformed) in N depleted cultures compared to N replete cultures (n = 3). Numbers indicate Phatr2 gene IDs. Phatr2_24186 and Phatr2_8113 were used as reference genes. Asterisks indicate significantly different expression in N depleted cultures compared to N replete cultures: *, p<0.05; **, p<0.01.

Supplementary Fig. S3

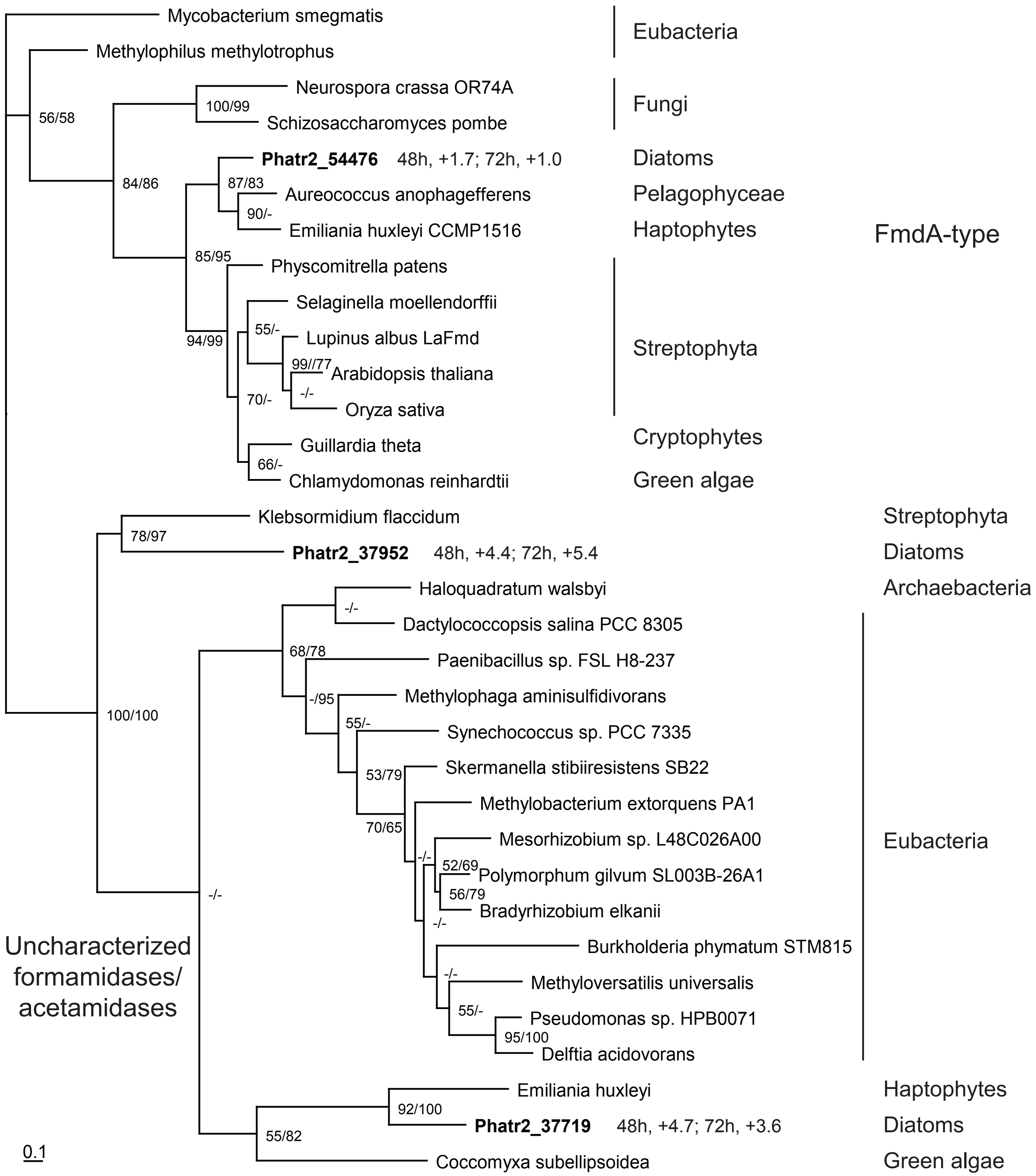

Fig. S3. Phylogenetic analysis of FmdA-type amidases/formamidases in *P. tricornutum*.

A Maximum likelihood (ML) tree was made based on a protein alignment of *P. tricornutum* FmdA-like amidases (shown in bold) with related amidases. Protein alignments were generated using Macaw 2.05 (NCBI) followed by manual refinement in GeneDoc 2.7.000 (Nicholas et al., 1999). ClustalX software (Larkin et al., 2007) was used to create bootstrapped neighbor-joining (N-J) (Saitou and Nei, 1987) trees using the Gonnet 250 score matrix. Bootstrapping of the N-J tree was performed with 1000 bootstrap trials. A number of substitution matrices were evaluated and the best one selected. Maximum likelihood trees were created with the RAxML program (version 7.2.6) using the GAMMA model of rate heterogeneity and the BLOSUM62 substitution matrix (Stamatakis, 2006). A total of 1000 non-parametric bootstrap inferences were executed. Trees were visualized using TreeViewX 0.5.0 (Page, 2002) and refined using Adobe Illustrator CS6.

The overall topologies for the ML and Neighbour joining (NJ) trees are the same. Bootstrap confidence values above 50% are shown in the tree, ML (first value) and NJ (second value). *Mycobacterium smegmatis* amdA was used as an outgroup. Relative gene expression values (log2 transformed) in N deprivated cultures compared to N replete cultures for each *P. tricornutum* gene is shown next to its gene ID. Accession numbers: *Arabidopsis thaliana* (NP_568029), *Aureococcus anophagefferens* (EGB06746), *Bradyrhizobium elkanii* (WP_026191996), *Burkholderia phymatum* STM815 (ACC74235), *Chlamydomonas reinhardtii* (XP_001699344), *Coccomyxa subellipsoidea* (XP_005644191), *Dactylococcopsis salina* PCC 8305 (YP_007170960), *Delftia acidovorans* (WP_016447502), *Emiliania huxleyi* CCMP1516 (XP_005759042 and XP_005788088), *Guillardia theta* (XP_005824474), *Haloquadratum walsbyi* (WP_021053649), *Klebsormidium flaccidum* (EST HO437218), *Lupinus albus* (ACM68705), *Mesorhizobium sp.* L48C026A00 (WP_023800326), *Methylobacterium extorquens* PA1 (YP_001638304), *Methylophaga aminisulfidivorans* (WP_007146869), *Methylophilus methylotrophus* (Q50228), *Methyloversatilis universalis* (WP_008063518), *Mycobacterium smegmatis* (Q07838), *Neurospora crassa* OR74A (XP_959782), *Oryza sativa* (NP_001044346), *Paenibacillus sp.* FSL H8-237 (ETT45786), *Phaeodactylum tricornutum* Phatr2_54476 (XP_002180029), Phatr2_37952 (XP_002182077), Phatr2_37719 (XP_002181627), *Physcomitrella patens* (XP_001775936), *Polymorphum gilvum* SL003B-26A1 (YP_004304071), *Pseudomonas sp.* HPB0071 (WP_010799697), *Schizosaccharomyces pombe* (Q9URY7), *Selaginella moellendorffii* (XP_002974928), *Skermanella stibiiresistens* SB22 (EWY38007.1), *Synechococcus sp.* PCC 7335 (EDX87519).

References:

Larkin M, Blackshields G, Brown N, Chenna R, McGettigan PA, McWilliam H, Valentin F, Wallace IM, Wilm A, Lopez R. 2007. Clustal W and Clustal X version 2.0. *Bioinformatics* **23**, 2947-2948.

Nicholas KB, Nicholas HBJ, Deerfield DWI. 1997. GeneDoc: analysis and visualization of genetic variation. *EMBNEW.NEWS* **4**, 14.

Page RD. 2002. Visualizing phylogenetic trees using TreeView. *Current Protocols in Bioinformatics* Chapter 6, Unit 6.2.

Saitou N, Nei M. 1987. The neighbor-joining method: a new method for reconstructing phylogenetic trees. *Molecular Biology and Evolution* **4**, 406-425.

Stamatakis A. 2006. RAxML-VI-HPC: maximum likelihood-based phylogenetic analyses with thousands of taxa and mixed models. *Bioinformatics* **22**, 2688-2690.

Supplementary Table S1. Tentatively identified algal metabolites based on GC–MS profiling. Values represent log2(n) ratios of DN to RN at sampling timepoints 48 h and 72 h. Color codes indicate concentration fold changes: increase - ■ 1.20-1.40, ■ 1.40-1.67, ■ 1.67-2.00, ■ >2 fold; decrease - ■ 1.20-1.40, ■ 1.40-1.67, ■ 1.67-2.00, ■ >2 fold.

COMPOND GROUP	METABOLITE	ratio DN/RN 48h	ratio DN/RN 72h	*p*-value
**Amines**	butylamine	-0.2382	0.7320	*0.0000*
	N-carboxy-methylamine	-0.6405	-0.4629	*0.0457*
	ethanolamine	-0.9413	-1.4001	*0.0000*
**Amino Acids**	alanine	-2.0909	-4.5802	*0.0000*
	sarcosine	-1.1894	-2.1188	*0.0000*
	valine	-2.1935	-4.5748	*0.0000*
	leucine	-2.3073	-2.7096	*0.0000*
	isoleucine	-3.0386	-3.4129	*0.0000*
	proline	-4.5951	-11.8100	*0.0000*
	glycine	-2.3093	-3.9808	*0.0000*
	serine	-2.0530	-2.8072	*0.0000*
	threonine	-1.2482	-1.3768	*0.0000*
	pyroglutamate	-4.3172	-5.2687	*0.0000*
	aspartate	-5.8538	-8.4446	*0.0000*
	glutamate	-4.0591	-6.6670	*0.0000*
	phenylalanine	-3.8788	-5.7818	*0.0000*
	asparagine	-3.5832	-4.6381	*0.0000*
	glutamine	-5.7464	-6.2234	*0.0000*
	ornithine	-6.2907	-6.6425	*0.0000*
	lysine	-4.8147	-4.1375	*0.0000*
	tyrosine	-3.8116	-3.7304	*0.0000*
	tryptophan	-4.1124	-4.7418	*0.0000*
**Fatty Acids**	C10:0	0.2752	1.1821	*0.0228*
	C12:0	-0.4521	-0.1399	*0.0137*
	C14:0	-1.7258	-1.4013	*0.0000*
	C15:0	-1.6099	-1.4381	*0.0000*
	C16:3 (9,12,15-(Z,Z,Z)-)	-0.0977	0.4221	*0.0148*
	C16:1 (9-(E)-)	-1.8887	-1.3091	*0.0000*
	C16:2 (7,10-(Z,Z)-)	-2.5049	-2.5109	*0.0000*
	C16:1 (9-(Z)-)	-1.7115	-1.8732	*0.0000*
	C16:0	-1.7270	-0.7859	*0.0000*
	18:2 (9,12-(Z,Z)-)	-1.4202	-0.1870	*0.7436*
	C18:1 (9-(Z)-)	-1.4769	-0.5287	*0.4367*
	C18:3 (9,12,15-(Z,Z,Z)-)	-0.9428	0.4353	*0.7774*
	18:2 (9,12-(E,E)-)	-0.9097	-0.2923	*0.7298*
	C18:1 (9-(E)-)	-1.6357	-0.6407	*0.2586*
	C18:0	-2.1435	-0.9069	*0.0001*
	C20:5 (EPA)	-3.0558	-3.2163	*0.0000*
	C20:0	-1.8188	-1.0792	*0.0575*

Supplementary Table S1 - continued

**Fatty Acids**	C22:6 (DHA)	-0.6594	-1.8389	*0.0064*
	C22:0	-2.0109	-1.5263	*0.0675*
	C23:0	-0.8581	-1.0878	*0.0748*
	C24:0	-1.9489	-2.0640	*0.0001*
	C25:0	-0.3399	0.1283	*0.8636*
	C26:0	0.4662	1.3154	*0.0219*
	C27:0	-0.3425	0.3059	*0.2382*
	C28:0	-0.7512	0.6008	*0.0812*
**Glycerides**	1-monomyristin	-0.6952	-1.2726	*0.0001*
	2-monopalmitolein	-1.4254	-1.4084	*0.0000*
	2-monopalmitin	-1.4576	-1.4706	*0.0000*
	1-monopalmitolein	-0.7204	-1.0386	*0.0000*
	1-monopalmitin	-0.5348	-1.0659	*0.0001*
**Organic Acids**	pyruvate	-2.0438	-3.7974	*0.0000*
	glycolate	0.2840	-0.6672	*0.6657*
	succinate	0.5500	-0.3347	*0.0036*
	glycerate	-0.5644	-1.4012	*0.0117*
	itaconate	0.1457	0.2433	*0.0020*
	fumarate	-0.0977	0.2139	*0.0000*
	2-methylmalate	-0.0597	0.2433	*0.0000*
	malate	-0.6616	-0.7207	*0.0002*
	threonate	-0.1087	-0.8036	*0.0000*
	2-oxoglutarate	-0.0977	-1.5029	*0.0000*
	(Z)-aconitate	-1.0596	-1.1466	*0.0399*
	citrate	-0.0977	-1.8474	*0.0000*
	dehydroascorbate	-1.0900	-1.7052	*0.0000*
**Phosphates**	phosphate	-2.4573	-3.9003	*0.0000*
	glycerol-3-phosphate	-3.8586	-6.3002	*0.0000*
	glycerophosphoglycerol	-3.8457	-4.6691	*0.0000*
	sugar phosphate_A221004	-2.2893	-4.0284	*0.0000*
	fructose-6-phosphate	-0.0977	-3.5026	*0.0000*
	glucose-6-phosphate	-0.8038	-3.6923	*0.0000*
	myo-inositol-2-phosphate	-0.0977	-2.7997	*0.0000*
**Sterols & Terpenes**	phytol, 2-(E)-	-0.5337	-1.4491	*0.0000*
	ergosta-4,6,22-triene	-1.0961	-0.9090	*0.0024*
	24-epibrassicasterol	-0.6894	-0.7211	*0.0000*
	cholesterol	-0.4511	-0.0622	*0.1992*
	ergosta-7,22-dien-3β-ol	-0.3421	-0.0982	*0.0000*
	campesterol	-1.7253	-2.7379	*0.0000*
**Sugars & Polyols**	glycerol	-0.0141	-0.5329	*0.0001*
	xylose	0.6786	-2.6967	*0.0000*
	arabitol	-1.8823	0.4413	*0.0000*
	fucose	-0.0977	0.2433	*0.0000*
	fructose	-2.8847	-2.3084	*0.0000*

Supplementary Table S1 - continued

**Sugars & Polyols**	glucose	1.1783	0.7505	*0.0001*
	mannose	-1.6313	-2.2010	*0.0000*
	galactose	1.0524	0.8025	*0.0001*
	myo-inositol	0.4810	1.0048	*0.0000*
	galactosyl glycerol	-1.3534	-1.9318	*0.0000*
	sucrose	-1.5573	0.2433	*0.2081*
	α,α-trehalose	0.6427	-0.0715	*0.0151*
	maltose	-0.6235	-0.7490	*0.0000*
	galactinol (isomer)	0.0535	1.2508	*0.0042*
**Others**	adenine	-0.0977	-0.6599	*0.0001*
	α-tocopherol	-0.8676	-1.2706	*0.0003*
